# Comprehensive analysis of retracted journal articles in the field of veterinary medicine and animal health

**DOI:** 10.1186/s12917-022-03167-x

**Published:** 2022-02-18

**Authors:** Mary M. Christopher

**Affiliations:** School of Veterinary Medicine, University of California–Davis, 4206 VetMed 3A, One Shields Ave, Davis, CA 95616 USA

**Keywords:** Editorial policies, Publication ethics, Publication misconduct, Research misconduct, Veterinary journals

## Abstract

**Background:**

Retractions are a key proxy for recognizing errors in research and publication and for reconciling misconduct in the scientific literature. The underlying factors associated with retractions can provide insight and guide policy for journal editors and authors within a discipline. The goal of this study was to systematically review and analyze retracted articles in veterinary medicine and animal health. A database search for retractions of articles with a veterinary/animal health topic, in a veterinary journal, or by veterinary institution-affiliated authors was conducted from first available records through February 2019 in MEDLINE/PubMed, Web of Science, Scopus, Retraction Watch, and Google Scholar. Annual frequency of retractions, journal and article characteristics, author affiliation and country, reasons for retraction, and retraction outcomes were recorded.

**Results:**

Two-hundred-forty-two articles retracted between 1993 and 2019 were included in the study. Over this period, the estimated rate of retraction increased from 0.03/1000 to 1.07/1000 veterinary articles. Median time from publication to retraction was 478 days (range 0-3653 days). Retracted articles were published in 30 (12.3%) veterinary journals and 132 (81.5%) nonveterinary journals. Veterinary journals had disproportionately more retractions than nonveterinary journals (*P* = .0155). Authors/groups with ≥2 retractions accounted for 37.2% of retractions. Authors from Iran and China published 19.4 and 18.2% of retracted articles respectively. Authors were affiliated with a faculty of veterinary medicine in 59.1% of retracted articles. Of 242 retractions, 204 (84.3%) were research articles, of which 6.4% were veterinary clinical research. Publication misconduct (plagiarism, duplicate publication, compromised peer review) accounted for 75.6% of retractions, compared with errors (20.6%) and research misconduct (18.2%). Journals published by societies/institutions were less likely than those from commercial publishers to indicate a reason for retraction. Thirty-one percent of HTML articles and 14% of PDFs were available online but not marked as retracted.

**Conclusions:**

The rate of retraction in the field of veterinary and animal health has increased by ~ 10-fold per 1000 articles since 1993, resulting primarily from increased publication misconduct, often by repeat offenders. Veterinary journals and society/institutional journals could benefit from improvement in the quality of retraction notices.

**Supplementary Information:**

The online version contains supplementary material available at 10.1186/s12917-022-03167-x.

## Background

Publication and research misconduct are important ethical concerns affecting the integrity of the biomedical literature [1–3]. Retraction of a published article is the primary means by which journals address and communicate scientific misconduct or errors. Retraction is a relatively rare event (estimated by some at ≤.02% [3]), but studies have demonstrated an increase in occurrence since about 2000-2001, especially in medical and life science disciplines [3–8]. This increase has been attributed in part to the increasing numbers of journals and scientific articles, which may exceed the capacity of the research community to provide adequate peer review [8]. The proliferation of open-access journals, some of which are low quality, has raised similar questions about peer review as well as whether editorial policies and oversight are adequate to prevent or appropriately handle publication misconduct. Increased retractions are also attributed to increased editor and publisher awareness of the retraction process, development of technological solutions to detect misconduct, shorter times between publication and retraction, and increases in some types of scientific misconduct. Some studies have found that journals with higher impact factors or highly cited articles are retracted more often [3]. Thus, multiple factors and variables can affect retraction rate.

A 2007 survey of science journal editors found that most editors were relatively unconcerned about publication ethics [9]. However, editors of self-published society or institutional journals may have fewer resources or be less aware of best practices for retractions than editors of journals published by commercial publishers. As the focus on publication ethics has increased, the Committee on Publication Ethics (COPE) published best practice guidelines for editors and journals on article retraction, including recommended content for retraction notices [10]. Retraction Watch, a mainstream blog that documents and comments on retractions in the literature, released its database in 2019, facilitating research on retractions [11]. Better understanding of the factors that lead to retractions and their implications for scientists and readers is key to maintaining the integrity of published research.

A focus on retractions by discipline draws attention to sources of error and ethical misconduct relevant to a specific research community and can guide discipline-specific education and mentoring in scientific research and publishing. Investigations into retractions have been reported for a wide range of biomedical disciplines and medical specialties, including human-subject research [12], cancer [13], surgery [14, 15], emergency medicine [16], dentistry [17], nursing [18], and radiology [19]. Veterinary medicine and animal health research is highly interdisciplinary, encompassing clinical and medical specialties; agriculture and animal science; basic and translational sciences; and wildlife medicine and conservation [20]. Veterinary medicine is also a relatively small field that may be difficult to assess as part of larger retraction studies, especially as veterinary clinicians and scientists publish both in veterinary and nonveterinary journals. A comprehensive review of retractions in veterinary medicine and animal health could be valuable for understanding the reasons, characteristics, and outcomes of retracted articles in the field and for improving processes that facilitate identification of errors and correction of the literature.

The goal of this study was to comprehensively review and analyze retracted articles in the field of veterinary medicine and animal health. The frequency of retractions, journal and article characteristics (including impact factor, topic, and species focus), author affiliations, reasons for retraction, and retraction outcomes and trends over time were determined. Additional goals were to compare publisher type, open-access journals, veterinary journals, and veterinary faculty-affiliated authors with other journals and authors. The results of this study will identify sources and trends in errors and in ethical misconduct (research and publication misconduct) and how they are reconciled in the veterinary and animal health literature, providing insight to publishers, journal editors, and authors, including veterinarians and animal health scientists.

## Results

**Fig. 1:**
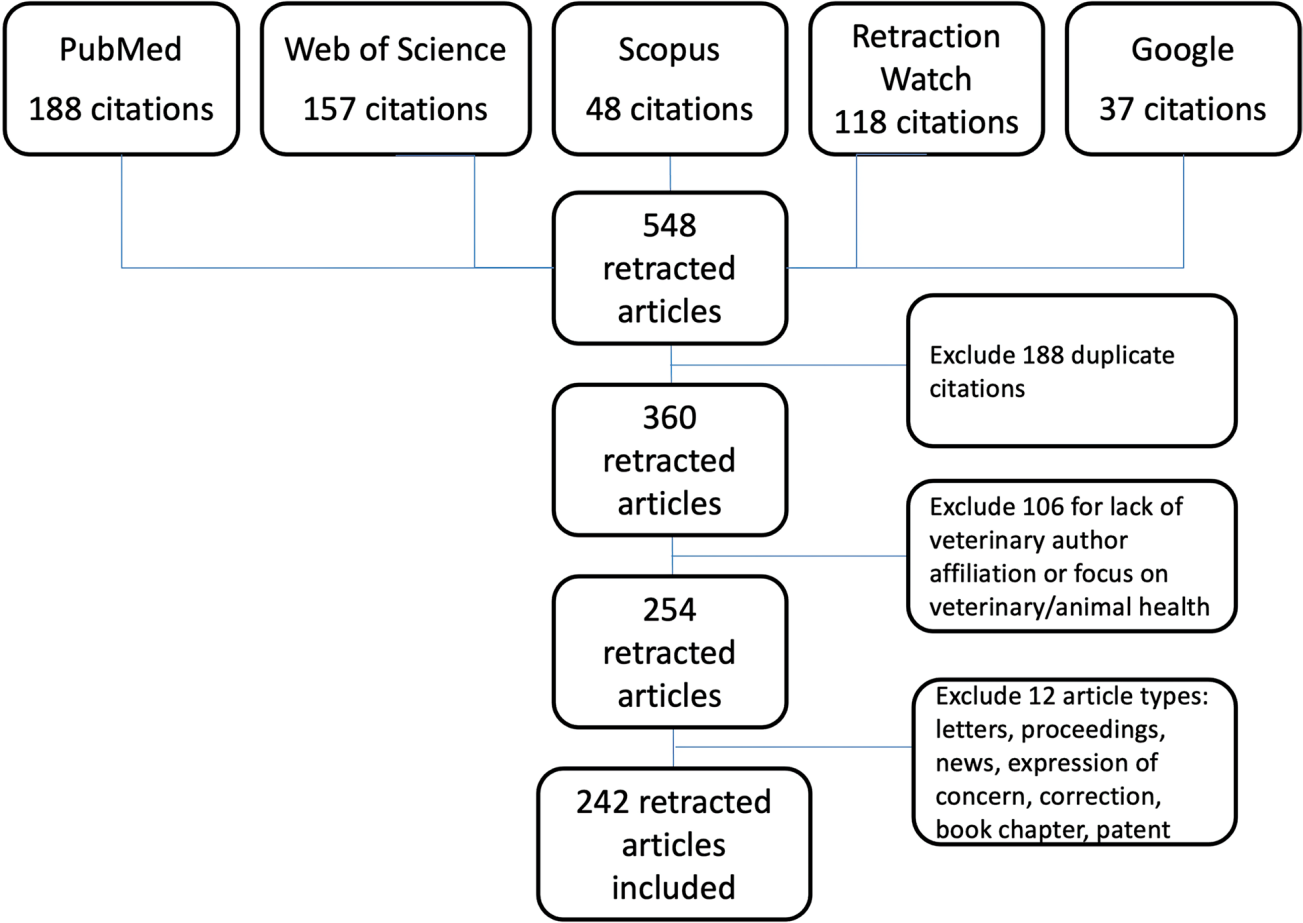
Search and retrieval process in a systematic review of retractions in the veterinary/animal health field

Of 548 citations retrieved, 306 were excluded and 242 were included in the study (Fig. 1). The 242 retracted articles involved veterinary medicine or animal health/disease (*n* = 188, 77.6%), were published in a veterinary journal (*n* = 57, 23.5%), and/or at least one author was affiliated with a veterinary faculty or a veterinary laboratory, department, or institute (*n* = 178, 73.5%). Two-hundred-twenty of 242 (90.9%) retracted articles were indexed in MEDLINE or were in PubMed Central, including 141 from the initial PubMed search and 78 retractions identified initially through other databases. Retractions not initially identified in PubMed occurred prior to 2013 when affiliations were included only for the first author; 12 retracted articles from other databases that were found in PubMed were not identified as retracted.

**Fig. 2:**
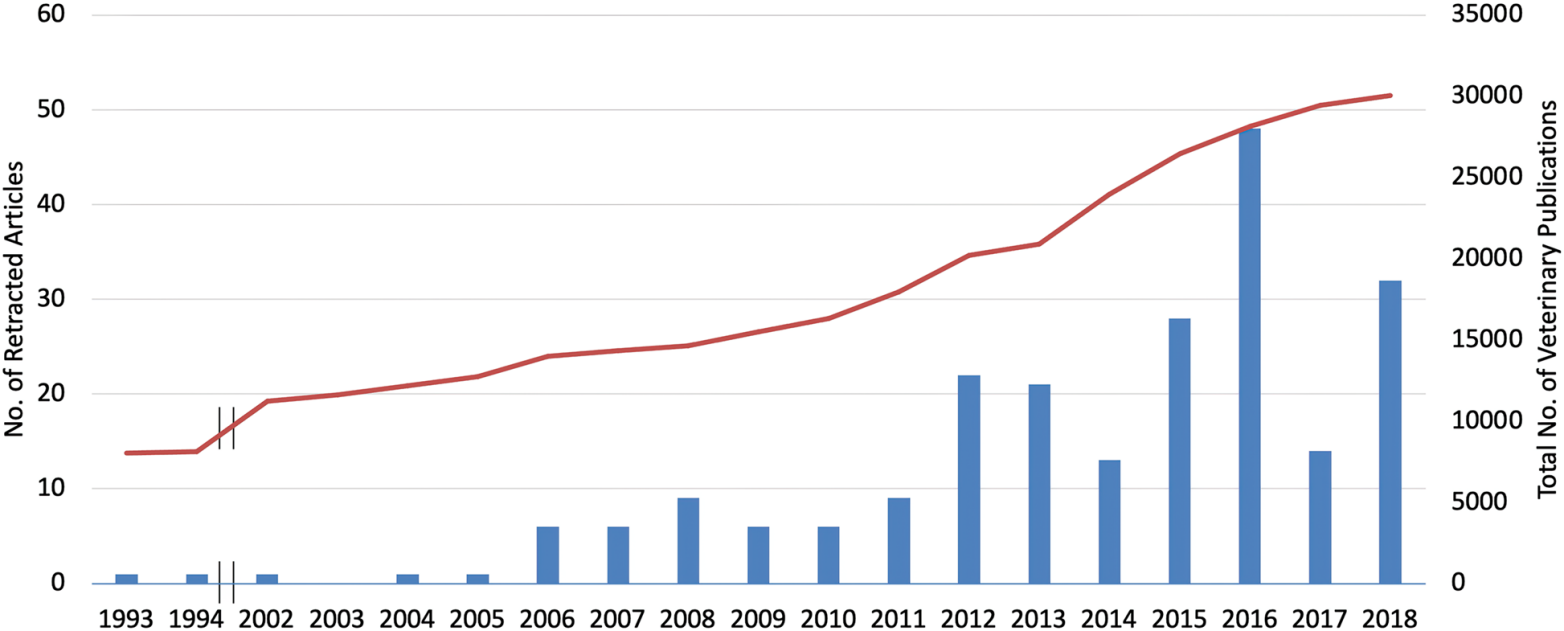
Total annual number of publications in PubMed using the search term “veterinary” (red line) and the number of retracted articles retrieved in this study (blue bars). No retractions were found from 1995 to 2001 or in 2003

Of the 242 database citations for retracted articles, 201 were labeled as retracted, 1 was labeled as partially retracted, and 40 were labeled as withdrawn. Withdrawn articles are generally defined as articles retracted while in press or after early online publication, but use of the term was sometimes inconsistent: eight articles identified as retracted in the database were identified at the journal or article level as withdrawn; and 10 articles identified as withdrawn were identified at the journal or article level as retracted. Year of retraction was based on the electronic retraction date (when available) or the print retraction date; a date of retraction was not found for 14 articles. Six of the 14 articles (42.8%) without a date of retraction were in unindexed journals found only in Google (of articles with a date of retraction, only 16/228 or 7.0% were not indexed). The number of retractions per year from 1993 through 2018 ranged from 0 to 48 (Fig. 2), with none found in 1995-2001 or in 2003. Three retracted articles identified in Jan-Feb 2019 were not included in annual frequency analyses. The annual number of retractions was < 10 in all years prior to 2012 and > 10 in all years after 2012, with a peak in 2016. Retraction rate was calculated using the total number of ‘veterinary’ articles in PubMed, divided into four year-groups, each with approximately equal numbers of articles. The frequency of retractions increased from 0.03/1000 (1993-2003) to 0.37/1000 (2004-2011) to 0.91/1000 (2012-2015) to 1.07/1000 (2016-2018), with an overall retraction rate of 0.56/1000 articles. Frequency was not calculated for 2019 because the full year was not represented.

### Journals and publishers

The 242 retractions were published in 162 unique journals published by commercial publishers, self-published by societies/institutions, or society journals hosted on a publisher website (Table 1). Thirty were categorized as veterinary journals by at least one major index. Veterinary journals had disproportionately more retractions than nonveterinary journals (*P* = .0155, Wilcoxon), accounting for 23.5% of retractions but only 12.3% of journals (Table 1). Forty-five (27.8%) journals were defined on their website as open-access. No significant difference was found in access or publisher type between veterinary and non-veterinary journals (*P* > .2505, Chi square).

**Table 1:** Journal characteristics for retracted articles

Characteristic	No. (%) Journals (***n*** = 162)	No (%) Retractions (***n*** = 242)
Indexed By*		
MEDLINE	128 (79.0)	200 (82.6)
PubMed Central	20 (12.3)†	20 (8.3)
Science Citation Index	134 (82.7)	ND
Scopus	149 (91.9)	ND
Journal Category		
Veterinary	30 (12.3)	57 (23.5)§
Nonveterinary	132 (81.5)	185 (76.4)
Publisher Type		
Commercial	110 (67.9)	160 (66.1)
Society/institutional	35 (21.6)	57 (23.6)
Commercial publisher-hosted societies	17 (10.5)	25 (10.3)
Accessibility		
Open-access	45 (27.8)	81 (33.5)
Traditional or hybrid	117 (72.2)	161 (66.5)

*Most journals were found in more than one index so percentages add up to > 100%. PubMed Central is not an index but is included here with MEDLINE to indicate the total retractions retrieved via PubMed

†Includes 14 complete journals and 6 with select citations, not in MEDLINE

§Significantly higher proportion than nonveterinary journals (*P* = .0155, Wilcoxon)

ND indicates not determined

Fifteen of 162 (9.2%) journals had ≥3 retractions each, accounting for 31.0% (75/242) of retracted articles (Table 2). Another 20 (12.3%) journals had 2 retractions each and 127 (78.4%) journals had 1 retraction each. A significantly higher proportion of veterinary journals (7/30, 23.3%) had ≥3 retractions compared with nonveterinary journals (8/132, 6.1%) (*P* = .0258).

**Table 2:** Journals with 3 or more retracted articles

Journal Title	No. (%) of Retracted Articles	Publisher	Veterinary Journal?	Open-Access?	2017 Impact Factor
Diagn Pathol	15 (6.2)^*^	Biomed Central	No	Yes	2.396
PLOS One	8 (3.3)^*^	Public Library of Science	No	Yes	2.766
Reprod Domest Anim	7 (2.9)^*^	Wiley	Yes	No	1.422
J Vet Med Sci	5 (2.0)	Japanese Society of Veterinary Science	Yes	Yes	0.803
J Vet Sci	5 (2.0)	Korean Society of Veterinary Science	Yes	Yes	1.327
Tumour Biol	5 (2.0)^*^	International Society of Oncology and BioMarkers (hosted by Springer)	No	No	–
Antioxid Redox Signal	4 (1.6)^*^	Mary Ann Liebert	No	No	6.540
J Biol Chem	4 (1.6)	American Society for Biochemistry and Molecular Biology	No	No	4.011
Vet Microbiol	4 (1.6)	Elsevier	Yes	No	2.525
Asian-Australasian J Anim Sci	3 (1.2)	Asian-Australasian Association of Animal Production Societies	No	Yes	–
J Anim Physiol Anim Nutr	3 (1.2)^*^	Wiley	Yes	No	1.607
J Clin Microbiol	3 (1.2)	American Society of Microbiology	No	No	4.054
J Med Primatol	3 (1.2)^*^	Wiley	Yes	No	0.432
J Parasit Dis	3 (1.2)^*^	Indian Society for Parasitology (hosted by Springer)	No	No	–
Res Vet Sci	3 (1.2)	Elsevier	Yes	No	1.616

^*^All or most retractions by “repeat offenders” (see Table 4)

One-hundred thirty-three of 162 (82.1%) journals had a 2017 impact factor, with a median of 2.476 (range 0.217–41.058). The median impact factor of veterinary journals (1.611, range 0.217-3.285) was significantly lower than for nonveterinary journals (2.734, 0.558-41.058) (*P* < .0001). No correlation or difference in impact factor was found among journals based on the number of retractions.

### Authors and affiliations

**Fig. 3:**
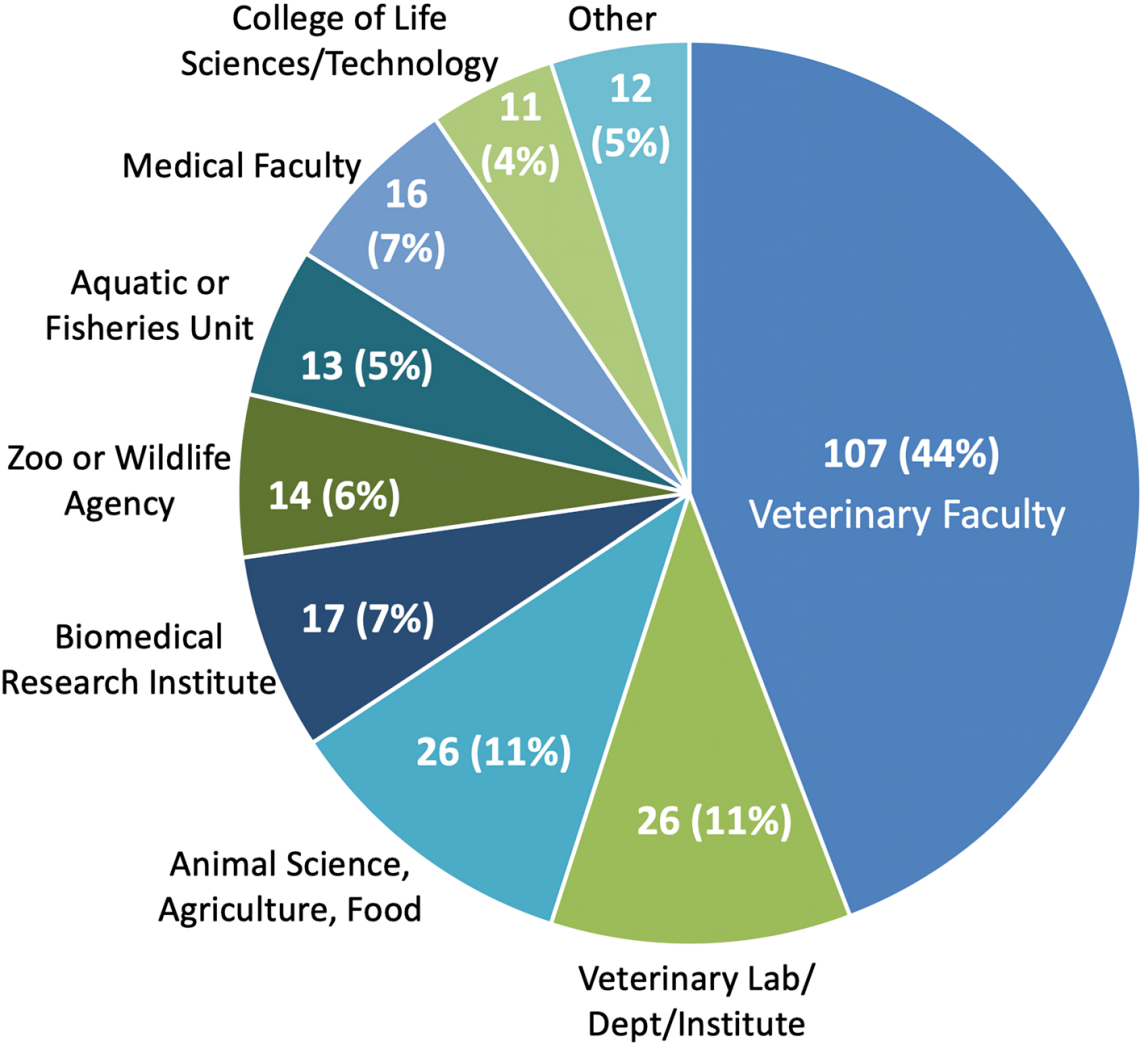
Institutional affiliation of the first author in 242 retracted articles in the veterinary/animal health field

A total of 143/242 (59.1%) retractions had faculty of veterinary medicine (FVM)-affiliated authors, of which 107/242 (44.2%) were first authors (Fig. 3). Retractions with FVM-affiliated (vs other) first authors were significantly more likely to involve companion animals, clinical research, animal models, and animal disease, and were less likely to involve fish or reproductive biology (*P* < .03). Fifty of 242 (20.6%) retractions had authors affiliated with a veterinary laboratory, department, or institute, of which 26 (52.0%) were first authors.

First authors were from 37 countries. Iran and China accounted for the most retractions, and together with other top 10 countries accounted for 78.1% (189/242) of retracted articles (Table 3). Three countries (Italy, Pakistan, Turkey) had 4 retractions each; 5 countries (Australia, Brazil, Gabon, Greece, Thailand) had 3 retractions each; 7 countries (Ethiopia, France, Nepal, Poland, Serbia, South Africa, Switzerland) had 2 retractions each; and 12 countries (Belgium, Canada, Croatia, Czech Republic, Finland, Norway, Philippines, Singapore, Sweden, Taiwan, United Arab Emirates, Vietnam) had 1 retraction each. Compared with other countries, a higher proportion of retracted articles from Spain (14/19) and Iran (39/47) were by authors or author groups with multiple retractions (*P* < .0001). The country affiliations of co-authors were similar to those of first authors, with 42 unique countries and 9 of the same 10 countries having the most retractions.

**Table 3:** Top ten countries with retracted articles based on the affiliation of the first author and the reason for retraction

Country	No. (%) of retracted articles	Reason(s) for Retraction*			
		Error	Research misconduct	Publication misconduct	Unknown
Iran	47 (19.4)	2	1	40†	3
China	44 (18.2)	8	4	26	8
Korea	19 (7.8)	3	6	8	2
Spain	19 (7.8)	2	15†	5	2
USA	18 (7.4)	10†	4	2	2
India	13 (5.4)	1	0	8	4
UK	10 (4.1)	5	1	2	2
Germany	7 (2.9)	1	2	3	1
Egypt	6 (2.5)	0	1	4	2
Japan	6 (2.5)	2	1	1	2

*Some articles were retracted for both research and publication misconduct, so numbers may add up to more than the total number of articles for that country

†Significantly different from other countries in the Table (*P* < .0001, Chi square); for Iran and Spain, these values include multiple retractions by the same author or author group (see Table 4)

Authors or author groups with 2 or more retractions (repeat offenders) accounted for 37.2% (90/242) of retracted articles (Table 4). Retracted articles by repeat offenders were overrepresented in journals with ≥3 retractions (44/75, 58.7%) and underrepresented in journals with only 1 retraction (29/127, 22.8%) (*P* < .0001). Retractions by repeat offenders accounted for a higher proportion of retractions due to publication misconduct (59/108, 54.6%) and a lower proportion of retractions due to errors (3/43, 7.0%) (P < .0001). A higher proportion of retractions by repeat offenders were published in nonveterinary (77/90, 85.6%) vs veterinary journals (13/90, 14.4%) (*P* = .0083).

**Table 4:** Authors and author groups with 2 or more retractions (repeat offenders)

Initials of Author/Group	Country	Faculty or Dept	No. Retractions /No. Journals	Reason(s) for Retraction	Formal Investigation Conducted
JJ et al. (40+ authors in var. combinations)	Iran	FVM	28/8	Compromised peer review, authorship irregularity, plagiarism	Internal investigation by a journal that published and retracted 15 of the articles
JAL	Spain	Natural Science Museum	12/7	Distrust data integrity/validity, authorship irregularity	External investigation by Spanish Superior Council of Scientific Research
MSA et al.	Iran	Dept of Fisheries	9/2	Compromised peer review	External investigation by university; internal investigation by two journals
SKK	Korea	FVM	5/2	Data fabrication/falsification	External investigation requested of author’s university
HK	Korea	Animal Science	5/4	Duplicate publication	Investigations by publisher (Korean Soc Animal Repro), ethical committee of journal
OP	UK	FVM	4/3	Found erroneous data/ analysis (author error)	None
NDA	Greece	Food Science	3/3	Duplicate publication	None
EM	Gabon	Medical Research Institute	3/1	Plagiarism	None
KPA	Nepal	Animal Science	2/2	Duplicate submission and publication	None
JLC-G^*^	Spain	Medical Faculty	2/1	Image manipulation	None
SC	China	State Lab of Vet Biotech	2/2	Distrust data integrity/ validity, authorship irregularity, plagiarism	None
WSH	Korea	FVM	2/1	Data fabrication/ falsification	External investigation by author’s university
SL	China	Vet Res Institute	2/2	Duplicate publication	None
ZXN, DL	China	FVM	2/2	Plagiarism	Internal investigation by affected journal
MAN	Iran	Animal Science	2/2	Duplicate submission & publication, plagiarism	None
FS, ZW	China	Animal Science	2/2	Plagiarism	External investigation by university of plagiarized investigator
RT^a^	Italy	Vet & Animal Science	2/2	Data fabrication/ falsification, image manipulation	External investigation by author’s university; internal investigation by one journal
MY	Pakistan	FVM	2/2	Plagiarism, authorship irregularity	No formal investigation but external inquiries into 1 article

^*^Additional articles published by this author/group were retracted from the literature, but the articles had insufficient relevance to the veterinary/animal health field and did not meet inclusion criteria for this study

FVM indicates Faculty of Veterinary Medicine

### Article types, topics, and species focus

Most (84.3%) retractions were research articles, with fewer case reports/case series and review articles (Table 5). Nearly half of retracted research articles involved basic research while only 6.4% involved veterinary clinical research. The most frequent topic of retracted articles was animal disease or animal disease investigations. Fourteen percent (35/242) of retracted articles had public health significance, with a focus on zoonotic disease, antimicrobial/drug residues, environmental toxicology, or food safety. Use of live animals (or their freshly harvested cells or tissues) was described in 181 retracted articles, including laboratory animals from institutional vendors (*n* = 76, 42.0%), free-ranging wildlife (*n* = 27, 14.9%), client-owned pets (*n* = 23, 12.7%), private or commercial farm animals (*n* = 16, 8.8%), institutional herds or colonies (*n* = 10, 5.5%), aquacultured fish (6, 3.3%), abbatoirs (*n* = 3, 1.6%), and zoos (n = 2, 1.1%). Twelve (6.6%) articles did not state the source of animals used. Six (3.3%) articles used human subjects or donors.

**Table 5:** Characteristics of retracted articles in the field of veterinary medicine/animal health

Characteristic	No. (%) of Retractions in Category	No. (%) of First Authors at FVM or Vet Dept/Lab/Institute
Type of Article (n = 242)		
Case reports/case series	20 (8.2)	17/20 (85.0)
Hypothesis	1 (0.4)	0
Research	204 (84.3)	105/204 (51.5)
Reviews	17 (7.0)	10/17 (58.8)
Type of Research (*n* = 204)		
Applied	39 (19.1)	15/39 (38.5)
Basic	92 (45.1)	51/92 (55.4)
Clinical (veterinary)	13 (6.4)	12/13 (92.3)
Clinical (human)	4 (2.0)	1/4 (25.0)
Epidemiology/field	31 (15.2)	11/31 (35.5)
Translational animal model	24 (11.7)	14/24 (58.3)
Unknown	1 (0.5)	1 (100)
Veterinary/Animal Health Topic (n = 188)		
Anatomy and physiology	14 (7.4)	9/14 (64.3)
Behavior and welfare	2 (1.1)	0 (0)
Disease/disease investigation	97 (51.6)	62/97 (63.9)
Food safety	10 (5.3)	5/10 (50.0)
Genetics (avian)	2 (1.1)	0 (0)
Nutrition	6 (3.2)	1/6 (16.7)
Pathology/microbiology/parasitology/immunology	27 (14.4)	11/27 (40.7)
Reproduction/reproductive biology	28 (14.9)	10/28 (35.7)
Toxicology	2 (1.1)	1/2 (50.0)
Public Health Relevance (*n* = 35)		
Food safety/drug residues/antimicrobial resistance	17 (48.6)	6/17(35.3)
Environmental toxicology	4 (11.4)	0/4 (0)
Zoonotic disease	14 (40.0)	6/14 (42.8)
Species Focus (*n* = 225)		
Avian (all but 1 wildlife)	19 (8.4)	2/19 (10.5)
Companion animal (dog, cat, horse)	35 (15.5)	30/35 (85.7)
Fish	17 (7.5)	3/17 (17.6)
Human	14 (6.2)	6/13 (46.1)
Laboratory (mouse, rat, rabbit)	47 (20.9)	33/47 (70.2)
Livestock (cattle, pig, sheep, goat, buffalo, camel)	52 (23.1)	31/52 (59.6)
Non-human primate	8 (3.5)	2/8 (25.0)
Non-domestic (bear, elephant, fox, hare, lynx, opossum, snake)	7 (3.1)	4/7 (57.1)
Multiple species	8 (3.5)	4/8 (50.0)
Poultry	18 (8.0)	10/18 (55.5)

### Retraction reasons and outcomes

The median time interval between publication and retraction was 477.5 days (range 0-3653 days, *n* = 228). Commercial publisher-hosted society journals had a significantly longer interval to retraction (median 789.5 days) compared with commercial publishers (243.5 days) and society/institutional publishers (472.5 days) (*P* = .0296). No significant difference in time to retraction was found for open-access or veterinary journals. Median days to retraction was significantly shorter for articles labeled withdrawn (0 days, range 0-1602 days) as compared with those labeled retracted (701 days, range 0-3653 days) (*P* < .0001). No correlation was found between the time to retraction and journal impact factor.

Reasons were provided for retraction in 207/242 (85.5%) articles (Table 6). The most frequent reason stated was publication misconduct, followed by research misconduct and errors (by the journal or by authors). Median time from publication to retraction was significantly shorter for retractions due to errors (273 days) as compared to publication misconduct (641 days), research misconduct (927 days), or both publication and research misconduct (1614 days) (*P* < .0001). A significantly higher proportion of veterinary journals did not state the reason for retraction (17/57, 29.8%) compared with nonveterinary journals (18/185, 9.7%) (*P* < .0001). A higher proportion of journals published by societies/institutions did not state the reason for retraction (16/57, 28.0%) compared with commercial publishers (19/160, 11.9%) (*P* = .0013). No difference was found in the reason for retraction between open-access and non-open-access journals. Median journal impact factor was significantly higher for articles retracted for research misconduct (4.011) compared with those retracted for errors (2.525) or publication misconduct (2.275) (*P* < .0001).

**Table 6:** Reasons for the retraction of articles in veterinary medicine/animal health

Category	Reason	No. (%) of Retractions^*^	Total No. (%) Retractions
Error			50 (20.6%)
	Administrative error by journal or publisher	13 (5.3%)	
	Erroneous data, analysis, or interpretation by author	37 (15.3%)	
Research misconduct			44 (18.2%)
	Data fabrication or falsification	23 (9.5%)	
	Suspected data fabrication/ falsification	5 (2.0%)	
	Image manipulation	13 (5.3%)	
	Research misconduct, not otherwise specified	3 (1.2%)	
Publication misconduct			183 (75.6%)
	Plagiarism, misappropriation of data	65 (26.8%)	
	Duplicate publication^†^	41 (16.9%)	
	Authorship irregularity	41 (16.9%)	
	Compromised peer review	36 (14.9%)	
Unknown			35 (14.4%)

^*^Retraction notices reporting both research and publication misconduct (n = 11) are counted in both categories, so percentages add up to > 100%

^†^Four of 41 retractions for duplicate publication also specified duplicate submission

**Fig. 4:**
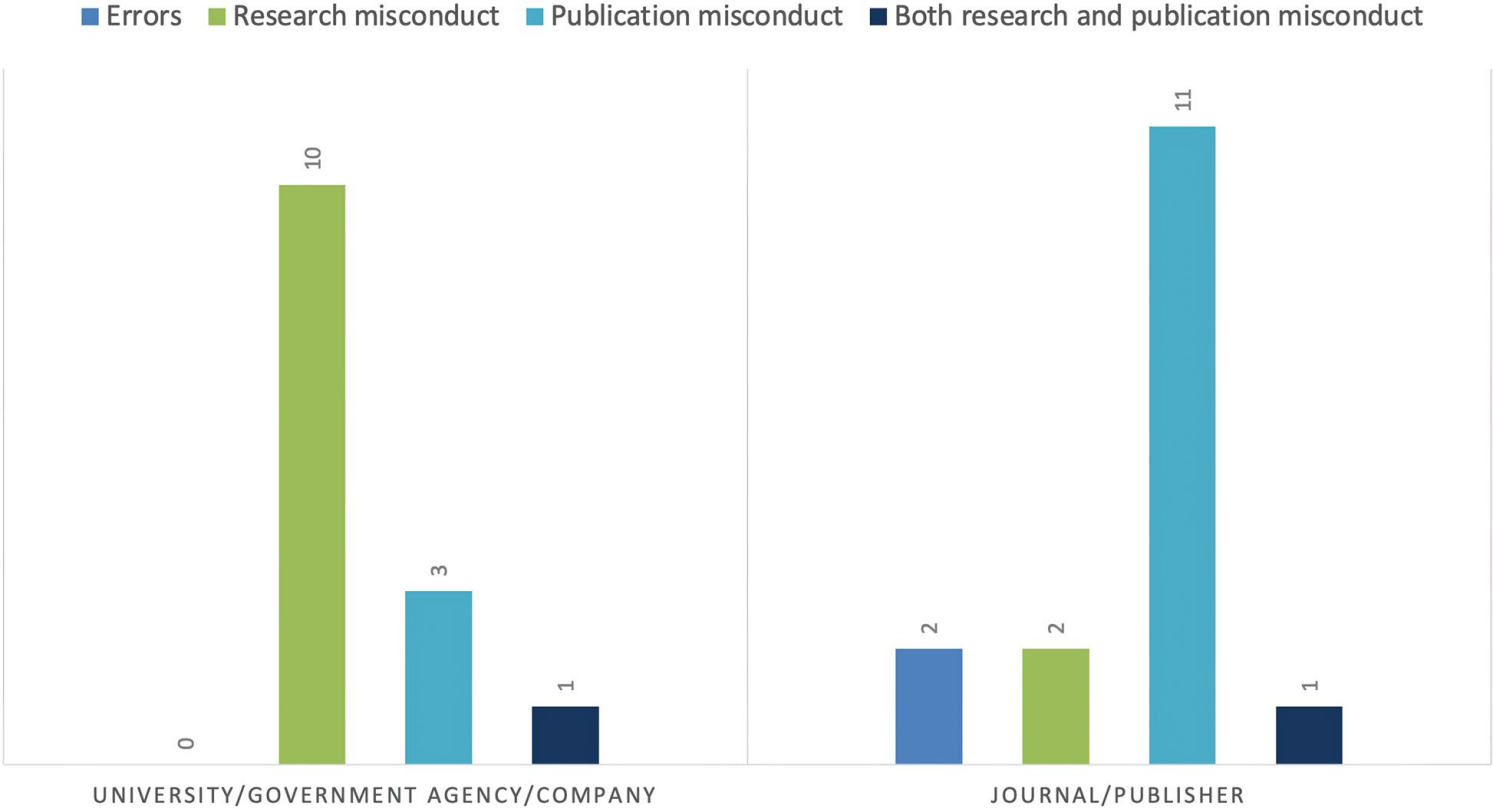
Investigations by institutions and by journals, based on the reason for retraction. Two cases of research misconduct and one case of publication misconduct involved investigations by both the journal and the author’s university

Formal investigations were conducted of 24 authors or author groups involving 65 retracted articles; 9 of the investigated authors were repeat offenders (Table 4). External investigations by universities (*n* = 11), government agencies (*n* = 2), and industry (n = 1) were conducted primarily in cases involving research misconduct; formal internal investigations by journals or publishers (7 veterinary and 7 nonveterinary journals) were conducted, primarily for cases of publication misconduct (Fig. 4).

The party initiating retraction was indicated in 227/242 (93.8%) of retraction notices; of these 14 indicated “author and/or editor” so the initiating party was not clear (Table 7). Authors, all or in part, initiated 43.3% (105/242) of retractions (including 91.4% of retractions involving errors); while editors and/or publishers initiated 44.6% (108/242) of retractions. A higher proportion of veterinary (7/53, 13.2%) vs nonveterinary journals (8/175, 4.6%) did not specify who initiated retraction (*P* = .0244). Similarly, a higher proportion of society/institutional-published journals (8/57, 14.0%) vs commercial publishers (7/146, 4.8%) did not specify who initiated retraction (*P* = .0021). Higher proportions of retractions were initiated by editors/publishers (vs authors) in open-access journals (58.0% vs 34.5%) and in cases of publication misconduct (62.6% vs 32.1%) (*P* < .01).

**Table 7:** Characteristics of retractions in the field of veterinary medicine/animal health

Characteristic	No. (%) of Retractions
Initiator of retraction (*n* = 242)	
Author(s)	59 (24.3)
Author and editor/publisher	46 (19.0)
Author and/or editor	14 (5.8)
Editor	45 (18.6)
Editor and publisher	39 (16.1)
Publisher	24 (9.9)
Not specified	15 (6.2)
Retraction notice on website (n = 242)	
Descriptive retraction notice	114 (47.1)
Link to descriptive retraction notice	68 (28.1)
Marked as retracted	17 (7.0)
No indication of retraction	43 (17.7)
Full-text HTML article availability and marking (n = 242)	
Available, unmarked	76 (31.4)
Available, marked as retracted (in title or watermark)	81 33.5)
Removed, title marked as retracted	51 (21.0)
Removed, no indication of retraction	34 (14.0)
PDF article availability and marking (n = 242)	
Available, unmarked	34 (14.0)
Available, watermarked	127 (52.5)
Available, includes retraction notice	10 (4.1)
Removed from journal website	67 (27.7)
Could not access (PDF behind paywall)	4 (1.6)

A majority of retracted articles (182/242, 75.2%) were accompanied by either a descriptive retraction notice or a link to a retraction notice. Society/institutional publishers were more likely than commercial publishers to simply state ‘retracted’ in the article title rather than provide a descriptive retraction notice (*P* = .0009). Forty-five percent (110/242) of HTML articles lacked any indication of retraction or had been removed from the website; 31.4% (101/242) of PDFs lacked any indication of retraction or had been removed from the journal website. A higher proportion of open-access journals (18/81, 22.2%) had unmarked article PDFs on the journal website compared with non-open-access journals (16/157, 10.2%) (*P* = .0234).

**Fig. 5:**
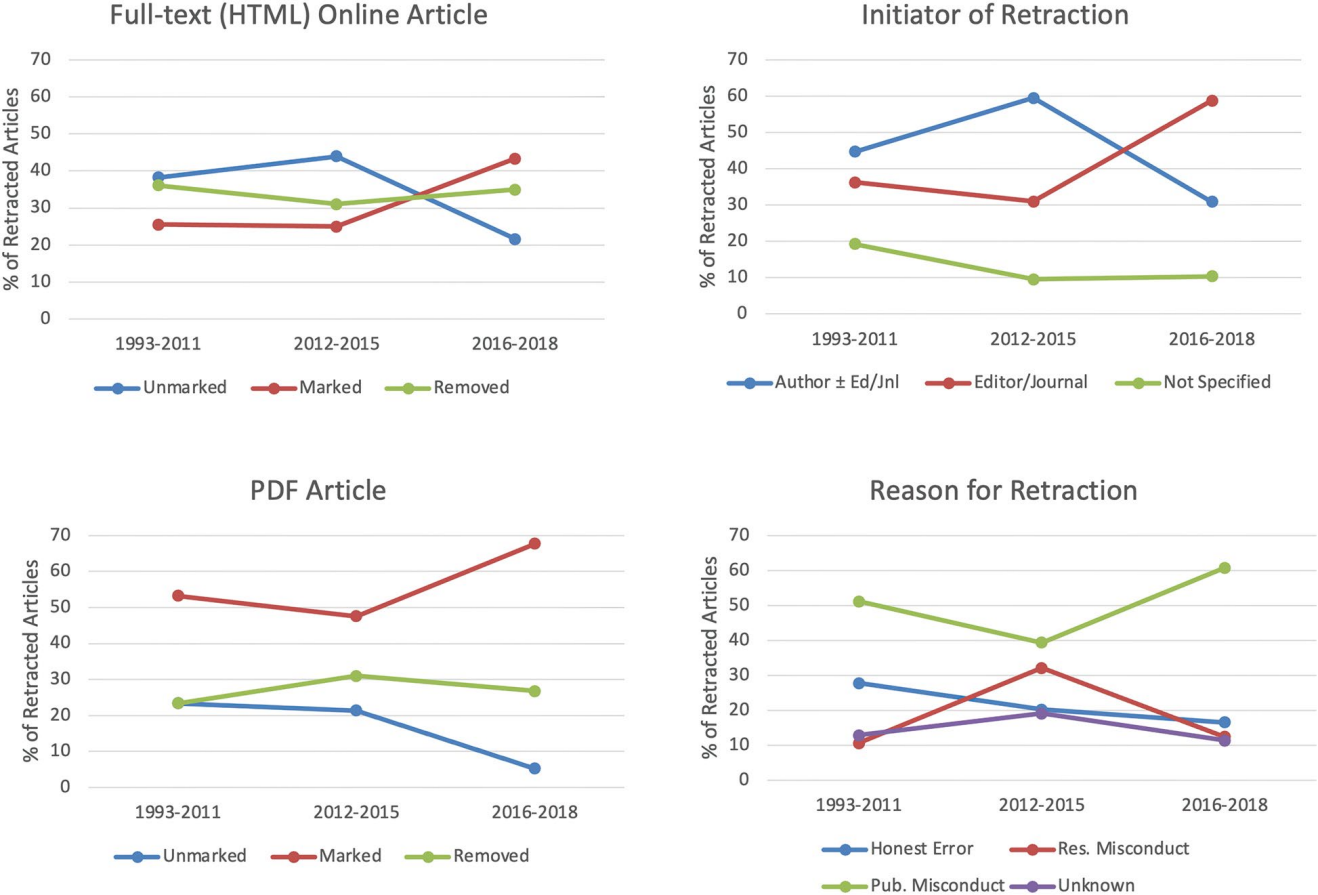
Trends over time in the outcomes of retracted articles in the veterinary/animal health field

Retraction outcome data were examined by year group (1993-2011, *n* = 47; 2012-2015, *n* = 84; 2016-2018, *n* = 94) for comparison of trends over time. The percentage of HTML and PDF articles marked as retracted increased significantly in 2016-2018 compared with prior years (*P* < .01) (Fig. 5). Editors/journals initiated a higher proportion of retractions in 2016-2018 compared with previous years (*P* = .0007). Significantly more retractions were the result of research misconduct (vs publication misconduct) in 2012-2015 (*P* = .0004). Median time from publication to retraction did not differ significantly in 1993-2011 (302 days) compared to 2012-2015 (409 days) and 2016-18 (686 days) (*P* = .1667).

## Discussion

Retractions in the field of veterinary medicine and animal health have increased substantively since 2012, with publication misconduct (especially plagiarism and duplicate publication) accounting for more than 75% of retracted articles. Notably, a few repeat offenders accounted for a high proportion of total retractions. In part because of this, veterinary journals had significantly more retractions than nonveterinary journals, and together with society/institutional journals, were less likely to provide informative retraction notices. These findings suggest that enhanced editorial oversight of peer review, and timely and transparent recognition of ethical misconduct, could help correct the literature and minimize the societal impact of retractions on the discipline.

Veterinary medicine and animal health is a diverse field that can be challenging to capture in bibliographic analyses. The comprehensive approach used herein included not only the veterinary journal category (across three major indexers for complete coverage), but also retractions with a topical focus on animal health and disease and those with authors at veterinary institutions, many of whom publish in non-veterinary journals. (CAB Abstracts also fully indexes veterinary topics but does not include retractions or retracted articles as a document type for searches so was not used in this study.) Unlike retractions included in the Veterinary Science category of Retraction Watch [21], the present study excluded laboratory animal studies intended solely to inform human health unless they included a veterinary-affiliated author. The result was a robust dataset that is widely representative of the field as well as the diverse work of veterinarians and those engaged in research in veterinary schools and research facilities. The main limitation of the dataset, as in other studies of retractions, was lack of a control group to compare with non-retracted article topics, authors, and journals. However, associations of these variables with the reasons for retraction and retraction outcomes were informative. Unlike larger bibliographic analyses [2, 4, 5], where veterinary science is inapparent or comprises a small, relatively unexamined subset of retractions, the present study expands our view of the field and thereby improves understanding of discipline-specific factors associated with retractions.

As reported in other bibliographic analyses [3–8], the rate of retraction in the veterinary medicine/animal health literature increased substantively over the study period, with a 10-fold increase (per 1000 articles) since 1993 and acceleration beginning in 2006, slightly later than for life and medical sciences in general [4, 5, 7]. Compared with some medical specialties (e.g., radiology and emergency medicine) [16, 19], the annual rate of retractions in veterinary medicine/animal health was relatively high, although other studies often used only a single database/index or had small samples sizes. A prevalence of 4/100,000 (1/25,000) was reported previously for veterinary science retractions, considerably lower than the estimated 50/100,000 (1/2000) prevalence reported here [2]. That study found only 17 retracted articles in veterinary science through 2011 (compared to 47 in that time period in this study), likely because article categories and prevalence were based solely on the Web of Science. Although PubMed veterinary publications were an imperfect denominator for calculating retraction prevalence, PubMed included a large majority of the retractions found across indexes and thus provided the best available estimate. Although an increase in retractions can result from increased efficiency in the system to correct the literature [6, 7], a temporal decrease in the time-to-retraction was not observed in the present study.

Retractions in veterinary medicine and animal health were most frequently the result of publication misconduct, which has been associated with reduced barriers to publication (e.g., lack of rigorous peer review) and inadequate editorial oversight. The proportion of retractions due to plagiarism and duplicate publication combined (44%) was similar to that reported in other studies and disciplines (42-48%) [5, 14, 16, 19, 22]. Other studies, on the other hand, have found research misconduct to be the primary cause of retraction [4, 12]. Compromised peer review was associated with two repeat offenders and 15% of retracted articles in the present study, a rate similar to that in a 2018 study of open-access journals [8]. Plagiarism and duplicate publication have increased since about 2005 [4], while compromised peer review has gained attention only more recently, since about 2015 [22, 23]. There is evidence of awareness of publication misconduct within the veterinary research community, with published exchanges in veterinary journals that range from apologetic [24, 25] to accusatory [26]. Such discussion also suggests a willingness to address and resolve issues of publication misconduct within the profession.

Veterinary journals comprised surprisingly few of the journals in this study, perhaps because of the predominance of basic (vs clinical) science articles retracted and because of the lower impact factors or perceived value of veterinary journals compared with other biomedical journals [27, 28]. Although high-impact journals and highly cited articles are retracted more often [3, 4], repeat offenders appeared to have had a disproportionate effect on the incidence of retractions in veterinary journals. Veterinary journals, together with self-published society/institutional journals, also had less informative retraction notices than other journals. These findings suggest weaker editorial processes or policies regarding publication misconduct, or a reluctance to acknowledge and address errors. Editors of small journals may be unaware of international publishing practices [29] and could benefit from stepped-up surveillance for potential misconduct (e.g., plagiarism) and improved retraction notices. That said, even high-impact journals seldom use plagiarism-checking services, and only a third provide authors with definitions of misconduct [30].

Author conduct and publication decisions are influenced by training, mentorship, affiliation, institutional culture, economic incentives, and national and academic ethical policies [2, 3, 23, 31]. In addition, retraction rates may track with overall publication rates, and although Western Europe and North America publish the majority of veterinary articles, publication rates from Asia (India, China), Latin America (Brazil), and the Middle East (Turkey, Iran) have notably increased since 2005 [20]. Authors of most retractions in the present study were from Iran and China, and the top 10 countries were similar to those of other studies [4, 5, 8, 31, 32]. The pattern of retractions differed, with Iranian authors often involving repeat offenders and peer review, while Chinese authors more often had 1 or at most 2 retractions, usually attributed to plagiarism, but also to errors. The latter finding differed from a study of retractions by Chinese researchers [33], which found a high incidence of repeat offenders and faked peer review. Had only authors with single retractions been considered in the present study, China would have ranked first, followed by the USA, India and Iran, and essentially the same top 10 list. India, which ranked 6th in retraction rate in the present study (5% of retractions), had the highest ratio of fraudulent to total papers in a study of low- and middle-income countries [31]. Plagiarism and duplicate publication are thought to stem, in part, from the root issue of originality, which can present a particular challenge for authors in some countries [32, 34].

Repeat offenders accounted for nearly 40% of retractions in veterinary medicine and animal health. Indeed, those with ≥5 retractions comprised nearly 25% of total retractions, more than double the 10% expected based on a power-law model of repeating probability [35]. This high proportion of habitual offenders introduced bias into analyses involving country of origin, author affiliation, and article topic. While sometimes viewed as anomalies or outliers [5], repeat offenders also provide unique insight into publication or research misconduct. Of particular note in this study was the Iranian group of repeat offenders. This group was reported previously to have 15 retractions in the online journal *Diagnostic Pathology* [8, 22]. In the present study, however, this group was associated with 28 retractions (11.5% of total) in 8 journals involving more than 40 authors in total. In addition, despite the FVM affiliation of many of the authors, none of the retractions was in a veterinary journal. Rather, the retractions included several case studies of common pathologic lesions or tumors in companion animals (e.g., mast cell tumors, mammary neoplasia) that have been well studied in the veterinary literature. Nonveterinary journals are more likely to lack appropriate veterinary content experts, both reviewers and editors, which could explain how the peer review process was repeatedly circumvented. Inadequate peer review practices are associated with retractions; specifically, closer involvement of the Editor-in-Chief and wider community in the review process is related to fewer retractions [36]. However, this alone may not be enough to prevent publication misconduct, as another Iranian repeat offender had multiple retractions in *Reproduction in Domestic Animals*, a veterinary journal appropriate for the research topic. The proliferation of low-quality, open-access journals can provide opportunity for repeat offenders to exploit new and unsupervised systems [8], but high-quality, open-access journals have also expanded, and the rate of retraction in open-access journals did not differ in the present study. Further, regardless of whether the peer review process was compromised by the authors, absent or inadequate peer review processes can occur if there is insufficient editorial board oversight and integrity.

Retractions reflect a failure to identify problems prior to publication but also signal a willingness on the part of journals and authors to correct mistakes [36]. Retraction notices are the primary means by which ethical breaches in research and publication are communicated to readers, although there is evidence that readers continue to use retracted information [37]. Just as importantly, retractions are intended to “correct” the literature and safeguard its integrity, although the lag from publication to retraction and differences among indexes can create inconsistency and initial confusion [10, 38]. Per COPE guidelines, retraction notices should be linked to all versions of an article; should clearly identify the retracted article; should clearly identify the action as a retraction; and should be published promptly [10]. Further, consistent language has been proposed for various forms of retractions and corrections [39]. Inconsistent usage of “retraction” and “withdrawal” was observed in a subset of the retractions in this study, and retraction notices were inconsistently linked to the HTML and PDF versions of articles.

It is recommended that a retraction notice also state the parties who initiated, issued, and supported the decision to retract; whether the authors were contacted and agreed to the retraction; the reason(s) for retraction; the sections retracted and the effect on the rest of the article; and the action taken by the journal [10]. Few retraction notices were complete in this regard and the considerable variation, as found in other studies, often made it difficult to ascertain the true cause of a retraction [3, 4, 12]. In some cases editors appeared to relitigate the peer review process and publish extensive external comments and complaints; in other cases articles simply disappeared without notice. Retractions initiated by authors due to errors in data, analyses, or reproducibility tended to have more detailed descriptions of how the integrity of the work was affected. The repeat offender from Spain in the present study was the result of distrust of data integrity and validity on the part of co-authors; retraction notices carefully described the part of the work affected, and those parts that remained credible. Notably in the current study, retraction practices were improved in 2016-18 compared with previous years, continuing a trend of improvement over time [40].

While the scholarly impact of articles and authors decreases after retraction, the societal impact of retractions on the field of veterinary science and public health can be substantive [4, 41]. Concerns involving contaminated animal feed and animal welfare, discussed extensively on Retraction Watch and in the media, have had significant implications for researchers and for the animal health industry [11]. The extensive external investigations initiated in a number of cases in the present study represent one of the more serious sanctions a journal can impose on offending authors [1]. A proposed system for maintaining the integrity of research (REPAIR) focuses on institutional and national responsibility, integrity, and transparency in the interest of creating a positive research culture [42]. Such practices will help maintain public confidence in the scientific literature.

## Conclusions

Retractions of journal articles in the veterinary medicine and animal health literature have increased substantially since 2012, primarily as a result of publication misconduct. Retracted articles reflect the breadth of animal health research and its diverse investigators, including both FVM and nonveterinary affiliations. Repeat offenders have a disproportionate effect on retractions, but recent trends suggest increasing editorial assertion and implementation of policies that better identify retracted literature. The societal impact of retractions on the animal health profession can be minimized through rigorous peer review processes, enhanced editorial oversight, use of digital tools, and the timely identification and transparent description of the error or misconduct.

## Methods

A systematic search for retracted articles was conducted in February 2019 using five databases: PubMed (MEDLINE and PubMed Central), Web of Science (Science Citation Index Expanded), Scopus, Retraction Watch, and Google Scholar. In PubMed, the Web of Science, and Scopus, a search on the term “veterinary” (all fields) was filtered by document type “retracted publications” and “retraction of publication” (and “withdrawal” for PubMed) for the entire time period of available articles (through February 2019). In Retraction Watch, retracted articles in the topic field “(HSC} Veterinary Science” were retrieved [21]. Google Scholar and Google were searched using variations on the terms “veterinary” and “retraction”; the first 25 pages of entries were examined or until 5 sequential pages yielded no relevant entries.

Retracted articles in English were included if they met at least one of the following inclusion criteria: (1) the article was published in a journal indexed as a “veterinary” journal by MEDLINE, Science Citation Index, or Scopus; (2) at least one of the authors was affiliated with a faculty (school/college) of veterinary medicine (FVM), or with a veterinary laboratory, department, or institute; and (3) the article involved veterinary medicine or animal health/disease (excluding invertebrates, but including fish and wildlife). When retraction notices listed additional articles by the same author(s) that had not already been retrieved in the search process, the additional articles were examined and added to the study if they met one or more of the inclusion criteria. Withdrawn articles, defined as articles retracted while in press or after early online publication, were included because of inconsistent and sometimes interchangeable use of the terms ‘retraction’ and ‘withdrawal’ by journals and indexers [39]. Articles were excluded if: (1) laboratory animals were used solely as a model of human disease and without a veterinary-affiliated author; (2) the author’s institutional affiliation included “Veterinary” only in a broader context (e.g., College of Medical, Veterinary, and Life Sciences) and otherwise lacked specific veterinary affiliation; (3) the term “veterinary” occurred solely in the cited literature of the article; and (4) the article was erroneously retrieved because it contained similar but irrelevant terms (e.g., “Veterans”). Retracted news articles, book chapters, commentary, corrigenda, and letters to the editor also were excluded.

Articles were given a unique identifier and the original citation and source (index) were recorded. PubMed was used as the core source; retraction records found initially in Web of Science, Scopus, Retraction Watch, and Google were subsequently also searched for in PubMed. Because more than 90% of retracted articles ultimately were found in PubMed, the PubMed dataset of “veterinary” articles was used to estimate the prevalence of retractions. Retracted articles, journal websites, and retraction notices were retrieved and examined. Data were recorded for variables associated with the journal, the article, and the retraction notice (Table 8). Journals were categorized as open-access if the journal website stated that it was an open-access journal, with all articles free to the public. Publisher type was categorized as commercial publishers, society/institutional publishers, and commercial publishers that hosted a society journal. Classification of article type (i.e., research, review, case report/case series) was based on article content. The affiliation and country of the first author and co-authors were recorded.

**Table 8:** Definition of variables in a systematic analysis of retractions in the field of veterinary medicine/animal health

Variables	Definition
Journals	
Journal title	–
Publisher	Commercial, society/institutional, commercial publisher-hosted society
Open-access	Yes/no
Indexing	MEDLINE, Science Citation Index (SCI), Scopus
Veterinary journal	Indexed in “VET” category in one or more indexes
Impact factor	Based on 2017 Journal Citation Report
Articles	
Publication date	Electronic and print publication dates
Article title	–
Authors	First author affiliation and country
	Co-author(s) affiliation and country
	Repeat offender (author or author group with ≥2 retractions)
Article type	Research, review, case report/series, other
Research type/topic	Basic, applied, clinical, epidemiology/field, translational model
	Veterinary/animal health topic (e.g., pathology, medicine)
	Public health relevance (yes/no)
	Animal species involved
	Live animal source (e.g., lab animal, wildlife, client-owned)
Retractions	
Retraction date	Electronic and print retraction dates
Time to retraction	Time interval between publication and retraction (days)
Reason for retraction	Error, research misconduct, publication misconduct
Initiated retraction	Author(s), editor, journal, publisher, combination
Investigation	Internal (journal) or external (institutional) investigation
Outcomes	HTML article: Available? Watermark? Retraction notice?
	PDF article: Available? Watermark? Retraction notice?

### Statistical analysis

Data were compiled in an Excel spreadsheet (Microsoft v 2019, Redmond, WA, USA) and analyzed statistically using JMP software (version 15.0, SAS Institute, Inc., Cary NC, USA). Based on visual assessment and the Anderson-Darling goodness-of-fit test, distribution of continuous variables (impact factor, days to retraction, number of retractions, number of authors) was found to be non-Gaussian. Therefore, those quantitative results were expressed as median and range (minimum-maximum values) and the nonparametric Wilcoxon rank sum test was used to compare results between groups. Categorical variables were expressed as frequency (%), and proportions were compared using Chi-square analysis. A *P* value of <.05 was considered as significant.

## Supplementary Information

**Additional file 1.** Retracted articles included in this study.

## Data Availability

The datasets used and/or analyzed during the current study are available from the corresponding author on reasonable request.
